# Cortisol Biosynthesis in the Human Ocular Surface Innate Immune Response

**DOI:** 10.1371/journal.pone.0094913

**Published:** 2014-04-15

**Authors:** Radhika Susarla, Lei Liu, Elizabeth A. Walker, Iwona J. Bujalska, Jawaher Alsalem, Geraint P. Williams, Sreekanth Sreekantam, Angela E. Taylor, Mohammad Tallouzi, H. Susan Southworth, Philip I. Murray, Graham R. Wallace, Saaeha Rauz

**Affiliations:** 1 Academic Unit of Ophthalmology, Centre for Translational Inflammation Research, College of Medical and Dental Sciences, University of Birmingham, Birmingham, United Kingdom; 2 Centre for Endocrinology, Diabetes and Metabolism, College of Medical and Dental Sciences, University of Birmingham, Birmingham, United Kingdom; Oregon Health & Science University, United States of America

## Abstract

Innate immune responses have a critical role in regulating sight-threatening ocular surface (OcS) inflammation. While glucocorticoids (GCs) are frequently used to limit tissue damage, the role of intracrine GC (cortisol) bioavailability via 11-beta-hydroxysteroid dehydrogenase type 1 (11β-HSD1) in OcS defense, remains unresolved. We found that primary human corneal epithelial cells (PHCEC), fibroblasts (PHKF) and allogeneic macrophages (M1, GM-CSF; M2, M-CSF) were capable of generating cortisol (M1>PHKF>M2>PHCEC) but in corneal cells, this was independent of Toll-like receptor (TLR) activation. While PolyI∶C induced maximal cytokine and chemokine production from both PHCEC (IFNγ, CCL2, CCL3, and (CCL4), IL6, CXCL10, CCL5, TNFα) and PHKF (CCL2, IL-6, CXCL10, CCL5), only PHKF cytokines were inhibited by GCs. Both Poly I∶C and LPS challenged-corneal cells induced M1 chemotaxis (greatest LPS-PHKF (250%), but down-regulated M1 11β-HSD1 activity (30 and 40% respectively). These data were supported by clinical studies demonstrating reduced human tear film cortisol∶cortisone ratios (a biomarker of local 11β-HSD1 activity) in pseudomonas keratitis (1∶2.9) versus healthy controls (1∶1.3; p<0.05). This contrasted with putative TLR3-mediated OcS disease (Stevens-Johnson Syndrome, Mucous membrane pemphigoid) where an increase in cortisol∶cortisone ratio was observed (113.8∶1; p<0.05). In summary, cortisol biosynthesis in human corneal cells is independent of TLR activation and is likely to afford immunoprotection under physiological conditions. Contribution to ocular mucosal innate responses is dependent on the aetiology of immunological challenge.

## Introduction

Ocular surface (OcS) pathology is a leading cause of worldwide blindness and represents a major proportion of ophthalmological emergencies in the developed world. The OcS provides an intricate mucosal barrier against infectious pathogens and immune-mediated processes that trigger innate responses. Unlike other mucosal surfaces the cornea is avascular and optically clear, and supports a highly complex tear film that is vital for lubrication, nutrition and immunological defence of the eye. The tear film ultrastructure comprises (i) a negatively charged, hydrophilic mucinous glycocalyx that limits adhesion of foreign debris or pathogens to the OcS, (ii) a more superficial nutritive aqueous phase consisting of a range of electrolytes, carbohydrates, anti-oxidants, vitamins, proteins, immunoglobulins, hormones, growth factors and cytokines that support epithelial cell proliferation, maturation and movement across the ocular surface, and (iii) a complex triplex lipid layer consisting of outer non-polar lipids, inner polar lipids with intercalated proteins together with a novel long chain (O-acyl)-ω-hydroxy fatty acid functioning as an intermediate surfactant lipid interface [1]. Immunological damage of the OcS results in mucosal inflammatory cell invasion, angiogenesis and ultimately a blinding keratopathy.

The tissue-specific regulation of the active glucocorticoid (GC), cortisol, is a purported mechanism for determining the length and type of inflammatory response [2]–[4]. The actions of GCs are largely mediated via the glucocorticoid receptor (GRα) that acts as a nuclear transcription factor for a variety of GC responsive genes and has been implicated in the pathogenesis of disease [5]–[8]. Systemic regulation of cortisol levels are controlled by the hypothalamic-pituitary-adrenal axis, but local regulation is mediated via the 11β-hydroxysteroid dehydrogenases (11β-HSDs). Two isoforms control the cortisol-cortisone shuttle [9]: 11β-HSD2 inactivating cortisol to cortisone [10], [11] in sodium and water secreting tissues protecting the mineralocorticoid receptor from GC excess, and 11β-HSD1 inducing GRα through activation of cortisol. 11β-HSD1 exhibits bidirectional enzyme activity *in vitro*, but *in vivo*, functions as an oxo-reductase, converting cortisone to cortisol - a reaction which is dependent on the delivery of co-factor NADPH via hexose-6-phosphate dehydrogenase (H6PD) and the glucose-6-phosphate transporter [12]. In the eye, our earlier work has localised 11β-HSD1 to the basal cells of the central corneal (differentiated) epithelium, and serum and glucocorticoid-regulated kinase 1 (SGK1, a GR mediated target gene) to the actively proliferating peripheral limbal region [13], [14]. The importance of GCs in maintaining the integrity of the corneal epithelium is further supported by analyses of cultured rabbit corneal epithelial cells (CECs)[3]. More recently we have shown that synthetic GCs, such as dexamethasone, are able to change the global gene and miR profile of corneal fibroblasts down-regulating inflammatory genes and inducing expression of anti-angiogenic and anti-inflammatory genes [15].

During pathogen-driven processes, the recognition of pathogen products occurs via receptors such as Nod-Like Receptors (NLR), NALPS/inflammasomes, RIG-I-like receptors (RLR) and Toll like Receptors (TLR) [16]. Several TLR have been shown to be expressed in the cornea with TLR 1–6 and TLR 9–10 in human conjunctival and limbal epithelial cells [17], and TLR3 in primary human corneal epithelial cells (PHCEC) [18]. The absence of expression of TLR7 and TLR8 in primary cells is of interest, as this suggests that PHCEC and primary human corneal fibroblasts (PHKF) do not respond to ssDNA and dsDNA through these receptors. TLR induction in these cells produce cytokines (IL-6, IL-8 and TNFα) but data are limited as no studies have examined a wider cytokine profile in order to interrogate disease signaling pathways in more detail. This is particularly relevant as TLR recognition of pathogen-derived products is also known to trigger an activating signal for the innate immune system during not only ocular infections, but also dry eye disease states and immune-mediated OcS disease such as Stevens-Johnson Syndrome/Toxic epidermal necrolysis (SJS-TEN) where ongoing sub-clinical inflammation remains a therapeutic challenge [16], [19]–[23].

In addition, macrophages and immature dendritic cells (iDCs) also respond to pathogens. Response may be direct via receptors such as the TLR or indirect, through cytokines and chemokines released by barrier cells under threat of the originating innate immune challenge agent. The autocrine regulation of 11β-HSD1 is pivotal; balancing the cortisol and cortisone shuttle appears to be a contributory driver of monocyte maturation, immune-cell function and down-regulation of tissue damaging inflammation [24], [25]. This could be particularly relevant in ocular disease where the optically clear media are paradoxically compromised by the innate immune response that is vital for eradication of the triggering agent.

In this study, we used a combination of *in vitro* and clinical studies to examine the interplay between TLR and GCs in the context of OcS health, and when the OcS is actively threatened by microbial insult or immune-mediated disease, to determine whether the local provision of GCs contributes to the innate immunity of the OcS in health and in disease.

## Materials and Methods

### Human corneal cells

#### Ethics Statement

The study was undertaken after formal ethics approval from the Black Country Research Ethics Committee (incorporating the Dudley Research Ethics Committee (LREC 06/Q2702/44)), and all experiments were carried out in accordance with the Tenets of the Declaration of Helsinki.

Donor peripheral corneal rims and central corneal buttons (transplant waste) from penetrating and lamellar keratoplasty surgical procedures, where the donor had given written informed consent for research were used for the culture of primary human corneal epithelial cells (PHCEC) and corneal fibroblasts (PHKF). According to United Kingdom Guidelines for Organ Donation, only those patients with normal eyes in the absence of absolute exclusion criteria including active transmissible disease or infection, Creuzfeldt-Jakob Disease, intravenous drug abuse, and neurodegenerative disorders, are suitable to donate organs and tissues for transplantation. Additional exclusions include previous ocular surgery, inflammation and tumors such as retinoblastoma.

The average age of the corneal donors was 69.1 (range 18–81.2) years. The corneal–scleral rims were incubated for 2 h at 37°C with 1.2 IU/ml neutral protease (Dispase II, Roche). Using a surgical ‘hockey stick’, the epithelium was stripped off with gentle scraping radially from the limbus towards the corneal apex in phosphate buffered saline (PBS). PHCEC were maintained in keratinocyte serum-free medium (KSFM) supplemented with 0.05 mg/mL bovine pituitary extract, 5 ng/mL human recombinant epidermal growth factor (Invitrogen, CA), 50 000 U/I penicillin and 50 000 µg/l streptomyocin, and 5% fetal calf serum (FCS) (Sigma, UK). Cells were grown at 37°C in a humid environment containing 5% CO_2_ and the cell culture media was changed every 2 to 3 days.

PHKF were generated from corneal tissue excised from the corneoscleral rims. The corneal epithelial layer was removed from the stroma using dispase enzyme digestion, and the endothelial layer stripped under direct visualisation with the aid of trypan blue. The remaining corneal stroma was chopped into small pieces and cultured in a 25 cm^2^ flask with 2 mL Fibro-medium. Cells were grown at 37°C in a humid environment containing 5% CO_2_ and the cell culture media was changed every 2 to 3 days. Confluent cells were trypsinised and only cells up to 4 generations were used for subsequent experiments.

The identity of the cell types were confirmed by immunocytochemical analyses for cytokeratin 3, anti-vimentin and anti-5B5 as previously described [15]. The PHCEC were identified by their hexagonal morphology and CK3^+^/vimentin^+^/5B5^−^ staining characteristics, whereas PHKF exhibited an elongated morphology and CK3^−^/vimentin^+^/5B5^+^.

### Monocyte-derived macrophages

Homogenous macrophage populations were generated by isolating peripheral blood mononuclear cells cells (PBMC) from the venous blood of healthy volunteers incubating pellets for at least 15 minutes in 20 µl of CD14^+^ beads (Miltenyi Biotech, UK) and 80 µl of MACS buffer (Miltenyi Biotech) per 1×10^7^/ml PBMC. Cells were then washed twice using MACS buffer and passed through a MACS column inserted into a magnetic holder. Negative subsets were excluded in the run through of column. The column was then removed from the holder and CD14^+^ cells were collected by washing and set up in complete media at an appropriate concentration. To grow M1 or M2 macrophages either 10 ng/ml of recombinant human GM-CSF (PeproTech, UK) or 50 ng/ml recombinant human M-CSF (PeproTech, UK), respectively, was added to purified monocytes in complete medium; RPMI 1640 media supplemented with 10 mM L-Glutamine, and 5000 U/ml penicillin with 10 mg/ml Streptomycin (GPS) with 10% heated inactivated fetal calf serum (HIFCS) (all Sigma UK) and 4% human serum (HD Supplies UK) and incubated at 37°C in a humidified atmosphere of 5% CO2 for 6–7 days and harvested for use.

### 11β-HSD enzyme assays

Cells grown to confluence were pre-treated with 10 ng/ml of cytokines (IL-1, IL6, IL-8, TNFα, IL-4, IL-10, IL-13; Peprotech,UK) for 24 h and 1 µg/ml LPS (Sigma UK) for 48 h in serum containing KSFM before the start of the assay. Dehydrogenase activity (cortisol to cortisone conversion) was assessed using 100 nM unlabelled cortisol (Sigma, UK) diluted in growth medium and tracer amounts (1.5 nM) of [^3^H]cortisol (specific activity 74.0 Ci/mmol; NEN, USA) at 37°C for 24 h. Conversion of cortisone to cortisol (oxo-reductase activity) was analysed by incubating cells with 100 nM cortisone and tracer amounts of [^3^H]cortisone (50000 cpm synthesised in-house) with or without cytokines. After 24 h incubation, steroids were extracted from the medium with ten volumes of dichloromethane, separated by thin-layer chromatography with chloroform∶ethanol (92∶8) as a mobile phase and the fractional conversion of steroids was calculated after scanning analysis using a Bioscan 2000 radioimaging detector (Bioscan, Washington, DC, USA). Following enzyme assay, cell monolayers were lysed in 1 ml water for subsequent protein assays. Total protein in each well was determined using a standard protein assay reagent (Bio-Rad), and enzyme activities were expressed as pmol/mg/h.

### RNA isolation and reverse-transcription (RT)

Total RNA was extracted from cells according to the manufacturer's instructions (Tri reagent, Sigma, UK) using. Total RNA was DNase I treated to remove any genomic DNA contamination (Invitrogen, CA). The quantity and quality of mRNA was assessed spectrophotometrically at and optical density of 260 /280 nm and by electrophoresis of 2 µl on a 1% agarose gel. Total RNA (100 ng-1 µg) in a total volume of 50 µl was reverse-transcribed using Multiscribe reverse transcriptase and random hexamers according to manufacturer's protocol (Applied Biosystems, UK). cDNA (50 ng) was used for conventional polymerase chain reaction (PCR) to define the expression of the genes of interest.

### Polymerase chain reaction (PCR)

RT was performed as above and 2 µl cDNA (PHCEC, PHKF) was used in subsequent PCR reactions for the following genes: 11β-HSD1; 11β-HSD2, GR, H6PD (PHCEC, PHKF, M1 and M2). The primer sequences, annealing temperatures and the PCR product sizes are detailed in **Table S1**. For the glucocorticoid signaling genes, the PCR cycling conditions consisted of 94°C for 5 mins followed by 35 cycles of denaturation at 94°C for 30 sec, annealing step for 30 sec, extension step 72°C for 30 sec and followed by heating at 72°C for 5 mins and storage at 4°C. The PCR products were analyzed on 2% agarose gel in 1xTBE buffer (0.089 M Tris-base, 0.089 M boric acid, 0.002 M EDTA, pH 8.3) and the DNA was stained with ethidium bromide and visualized with UV light.

### Cytokine analysis

PHCEC and PHKF cells at 90% confluence were treated with TLR1-9 ligands (Invivogen) (San Diego, CA, USA) for 16 h with cell culture medium only as negative control. The various TLR ligands and their concentrations used were: TLR1/2 (Pam3CSK 4 1µg/ml), TLR2 (HKLM 10^8^ cells/ml), TLR3 ((Poly(I∶C) 10 µg/ml), TLR4 (LPS 1µg/ml), TLR5 (Flagellin 1 µg/ml), TLR6 (FSL1 1 µg/ml), TLR7 (Imiquimod 1 µg/ml), TLR8 (ssRNA40 1µg/ml) and TLR9 (ODN2006 5 µM).

Cell supernatants were collected and multiplex bead assay analyzer (Luminex 100; Luminex Corporation, Austin, TX) was performed with a human cytokine 30-Plex kit (Invitrogen, Camarillo, CA) according to manufacturer's protocol and as previously described [26]. The panel included EGF, Eotaxin, FGF-basic, G-CSF, GM-CSF, HGF, IFN-α, IFN-γ, IL-1RA, IL-1β, IL-2, IL-2R, IL-4, IL-5, IL-6, IL-7, IL-8, IL-10, IL-12p40/p70, IL-13, IL-15, IL-17, CCL2, CXCL9, CCL3, CCL4, CCL5, TNF-α, and VEGF.

### Monocyte migration assays

PBMC were prepared from fresh whole blood sample from healthy volunteers by Ficoll-Paque PLUS (GE Healthcare Life Sciences, Amersham, UK). Briefly, whole blood was diluted by mixing with equal volume of RPMI containing 50 000 U/L penicillin, 50 000 µg/L streptomycin and 1% (v/v) Hepes. Diluted blood sample was carefully layered onto the Ficoll-Paque PLUS and centrifuged at 1200 rpm for 30 min at 20°C. PBMC was carefully collected at the interface between the Ficoll-Paque PLUS and sample layers. PBMC was washed with RPMI for 5 times.

Migration assays were performed with a 48-well Microchemotaxis Chamber (Neuro Probe, Inc., Gaithersburg,USA). The supernatants of LPS and Poly I∶C treated PHKF and PHCEC at volume of 26 µL were loaded in the bottom wells. A slight positive meniscus formed to prevent air bubbles from being trapped when the filter membrane was applied. PBMC suspension of 50 µL volume at concentration of 1×10^6^ cells/mL was loaded in the upper wells. The Chamber was incubated at 37°C in humidified air with 5% CO_2_ for 90 min. The non-migrated cells on the top side of the filter were wiped off and washed with PBS in a container. The filter was stained in Diff-Quick staining kit (IMEB, San. Marcos, CA) and mounted in immersion oil on a slide. Migrated cells were counted in three random 400× fields by two observers and averages of migrated cell number were calculated.

### Immunohistochemistry

Immunohistochemical analyses for the expression of TLR3, TLR4 and CD68 were performed on formalin-fixed, paraffin-embedded tissue sections of normal human anterior segments enucleated for posterior segment pathologies, and from herpetic keratitis and gram-negative keratitis tissue sections (6 µm). Antigen retrieval was performed by heating the slides at 95°C for 1 h in 10 mM sodium citrate buffer, pH 6.0 and subsequent cooling in cold sodium citrate buffer for 20 min. Sections were incubated with primary antibodies at 1;100 for anti-human TLR3, TLR4 and CD68 in 1% normal donkey serum overnight at 4°C. Following washing in PBS, biotin-conjugated secondary antibody was diluted in 1% blocking serum and and added to sections for 45 min at room temperature at dilutions of 1∶250 (donkey anti-sheep HRP conjugate) and 1∶150 (anti-rabbit). The detection was carried using DAB substrate (Dako, Cambridge, UK) and nuclei were counterstained using Meyer's Haemalum (Lamb Ltd, UK). Sections incubated in secondary antibody alone were used as controls.

### Quantification of hormones in human ocular biofluids

Tears (10 µl), aqueous humor (AqH –50 µl) and serum samples (100 µl) were collected from 43 healthy subjects prior to cataract surgery, and tears and serum from patients with either culture positive gram negative bacterial (pseudomonas) keratitis (n = 6) or PCR confirmed herpes simplex viral epithelial keratitis (n = 7) presenting to the Accident & Emergency Department at the Birmingham & Midland Eye Centre before initiation of treatment or instillation of eye drops. Tears and serum were also collected from patients with clinically quiescent chronic immune-mediated ocular surface disease (OcSD) for a minimum of 3 months who were not using therapeutic glucocorticoids (OcSD: Ocular Mucous Membrane Pemphigoid (OcMMP; n = 8); SJS-TEN; n = 5). Steroids were identified and quantified using liquid chromatography with tandem mass spectrometry (LC/MS/MS) which combines the physical separation capabilities of liquid chromatography with the sensitivity of mass spectrometry (Waters Xevo with Acquity uPLC), as previously described [27], [28]. Each steroid was extracted by liquid/liquid extraction and quantified by comparison to a calibration series with respect an internal standard, yielding quantification of unbound ‘free’ steroid hormone. Data were converted to ng/ml and presented in nM.

All human samples were taken after written informed consent approved by the Birmingham East, North and Solihull (BENS) Research Ethics Committee (LREC 08/H1206/165) and from the Black Country Research Ethics Committee (incorporating the Dudley Research Ethics Committee (LREC 06/Q2702/63).

### Statistical analysis

Statistical analysis was performed using the software package SigmaStat (Systat Software, USA.) and Prism for Windows version 4.03c (GraphPad Software, Inc., San Diego, CA, USA). One-way ANOVA with Dunnett's multiple comparisons post-test (for comparisons between untreated control and treatments) and Two-way ANOVA with Bonferroni post-test (for comparisons between groups of treatments) for analyses involving primary culture data. Due to donor variability in the cytokine production, the data have been normalized to respective untreated control values and presented as Mean ± SE, n = 3 donors. For biofluid hormone levels, the data are presented as median and ranges and analysed using two-tailed Mann-Whitney post–test for comparisons between group, and nonparametric Spearman-Rank correlation. P values <0.05 were accepted as statistically significant.

## Results

### Human corneal epithelial cells and corneal fibroblasts produce cortisol

**Figure 1:**
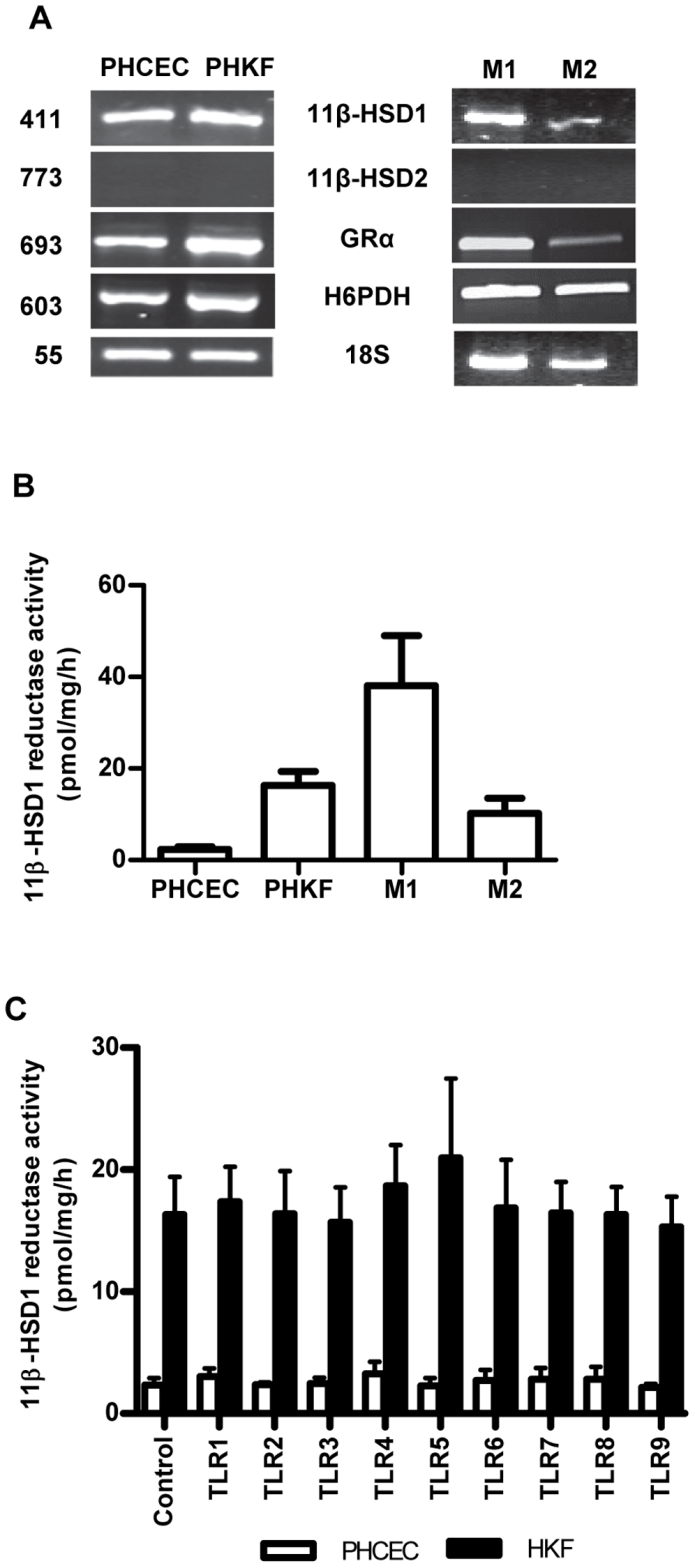
Pre-receptor regulation of glucocorticoids and TLR expression in human corneal cells. (A) RT-PCR of PHCEC, PHKF showing expression of the major genes for the pre-receptor regulation of glucocorticoids: 11β-HSD1, 11β-HSD2, H6PD and GR. Macrophages, M1 and M2, also express 11β-HSD1 but not 11β-HSD2. All cell types demonstrate 11β-HSD1 oxo-reductase activity (B), most marked in M1 macrophages. (C) TLR 1–9 induction did not alter 11β-HSD1 activity in either PHCEC or PHKF after stimulation for 16 h.

All cell types (PHCEC, PHKF, M1 and M2 macrophages) showed expression of 11β-HSD1, H6PD and GRα mRNA, whereas no cell type expressed 11β-HSD2 (**Figure 1A**). To confirm whether cells were capable of generating cortisol, oxo-reductase activity assays were performed by incubating cells with tritiated cortisone and conversion to cortisol was measured by using thin-layer radio-chromatography. All cell types produced active cortisol (**Figure 1B**), but activity was greatest for M1 macrophages (38.1±10.1 pmol/mg/h) followed by PHKF (16.3±3.1 pmol/mg/h), M2 macrophages (10.2±3.3 pmol/mg/h) then PHCEC (2.3±0.6 pmol/mg/h). There was no dehydrogenase activity in each of the cell types. Although expression of TLR mRNA was confirmed in these cells (**Figure S1**) as previously reported, TLR 1-9 agonism did not affect 11β-HSD1 mediated cortisol production in either of the corneal cell types (**Figure 1C**).

### TLR induction of cytokines by human corneal epithelial cells and fibroblasts

**Figure 2:**
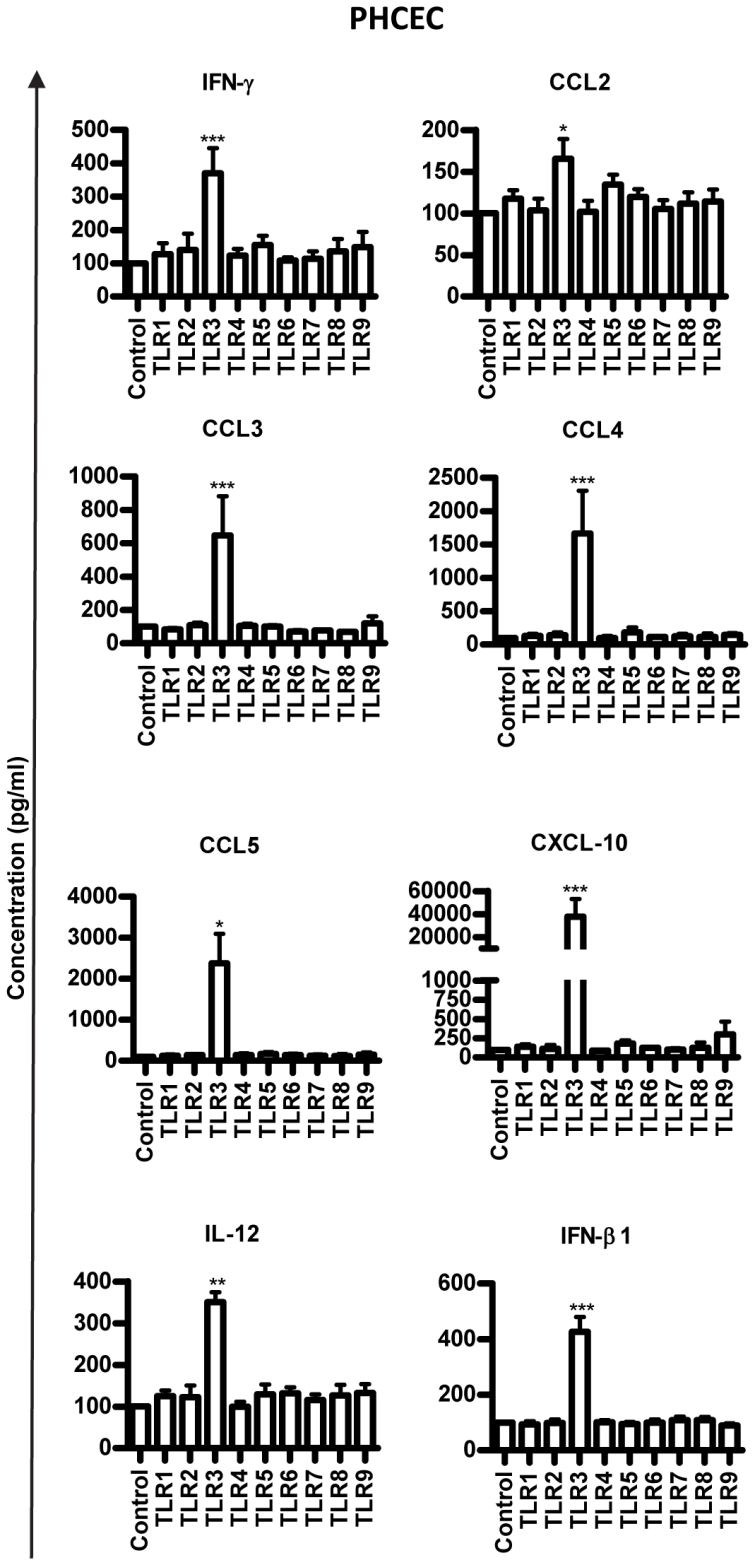
Human corneal cells respond to TLR stimulation by producing cytokines. Multiplex bead ELISA analysis (Plex-30) of cytokine production in response to TLR 1–9 stimulation for 16 hours is shown. Cytokines analysed included: IL-1β, IL-1Rα, IL-2, IL-4, IL-5, IL-6, IL-7, IL-8, IL-9, IL-10, IL-12(p70), IL-13, IL-15, IL-17, Eotaxin, Basic FGF, G-CSF, GM-CSF, IFN-γ, CXCL-10, CCL2, CCL3, CCL4, PDGF-BB, CCL5, TNF-α, VEGF. Induction of a range of cytokines and chemokines was seen after TLR 3 challenge (CCL2, CCL3, CCL4, CCL5, CXCL10, IL1, IFNγ and IFNβ). Values =  Mean+SE, n = 3, Statistical analysis was carried using One-way ANOVA and comparisons were drawn with untreated control Vs TLR stimulated cells. * p<0.05, ** p<0.01, *** p<0.001.

As previous literature had analyzed only specific cytokine production by these cells after TLR stimulation, we wished to address a wider cytokine profile using multiplex technology in order to interrogate GR signaling pathways in more detail. Cells were incubated with TLR ligands 1–9 and a “30-plex” multiplex analysis for cytokine and chemokine production was performed. Although synthesis of TNFα increased after TLR 1, 4, 7, and 9 stimulation of PHCEC this was not significant (**Figure S2**), and the epithelial cells were relatively specific to TLR3 challenge only, producing a large spectrum of cytokines and chemokines (CCL2, IL6, CXCL-10, IL12, CCL3, CCL4, IFNγ, IFNβ, and CCL5) (**Figure 2A**). By contrast, PHKF were weakly responsive to TLR stimulation showing increased expression of CCL2, IL-6, CXCL8, G-CSF CCL5 and CXCL-10. However none of these responses reached significance. (**Figure S2**).

### Glucocorticoid control of cytokine production by corneal cells

**Figure 3:**
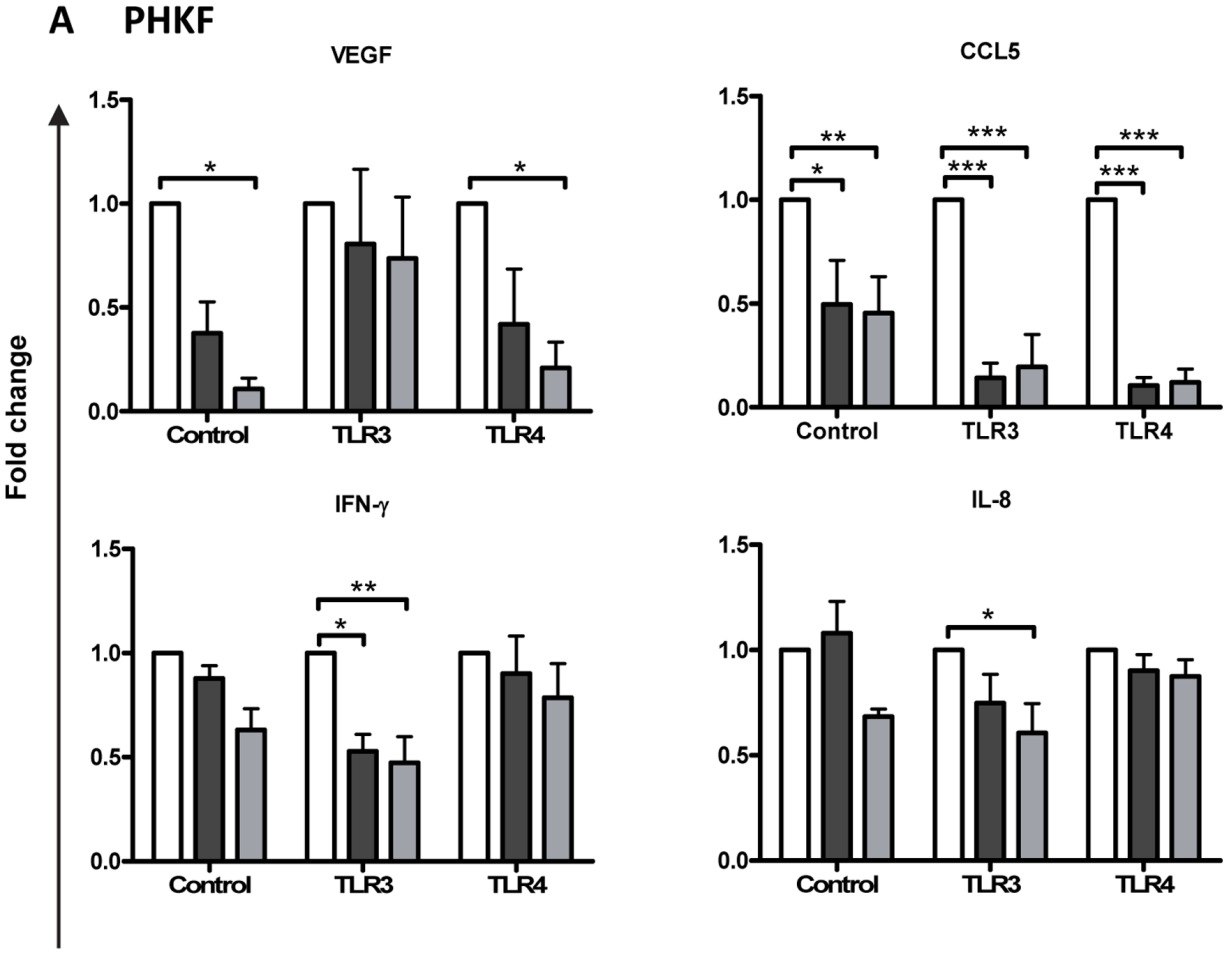
Regulation of cytokine production with Cortisol/Dexamethasone on TLR3 or TLR4 stimulated Primary Human Corneal Fibroblasts (PHKF). Both cortisol (▪, black square) and dexamethasone (▪, gray square) reduced cytokines: VEGF, CCL5, IFN-γ, CXCL-10, IL-8 and GCSF (B) after either or both TLR 3 and 4 stimulation of PHKF. (Values =  Mean+SE, normalising to No Cortisol/Dexamethasone (□, white square) for each treatment n = 3; Statistical analysis 2-way ANOVA with Bonferroni post-test; *p<0.05, **p<0.01, ***p<0.001).

Having identified that TLR3 and TLR4 stimulation of PHCEC and PHKF produced maximal production of most cytokines and chemokines we went on to assess the effects of natural and synthetic glucocorticoids (cortisol and dexamethasone respectively) on this process using multiplex bead analysis. The results are expressed as fold change to compare the two treatments with controls. Both cortisol and dexamethasone inhibited VEGF, CCL5, IFNγ, CXCL-10, CXCL8 and G-CSF production by PHKF (**Figure 3**) but no change in the other cytokines measured. There was no change any of the measured cytokines in PHCEC. (**Figure S3**)

### Monocyte/Macrophage infiltration in human keratitis

**Figure 4:**
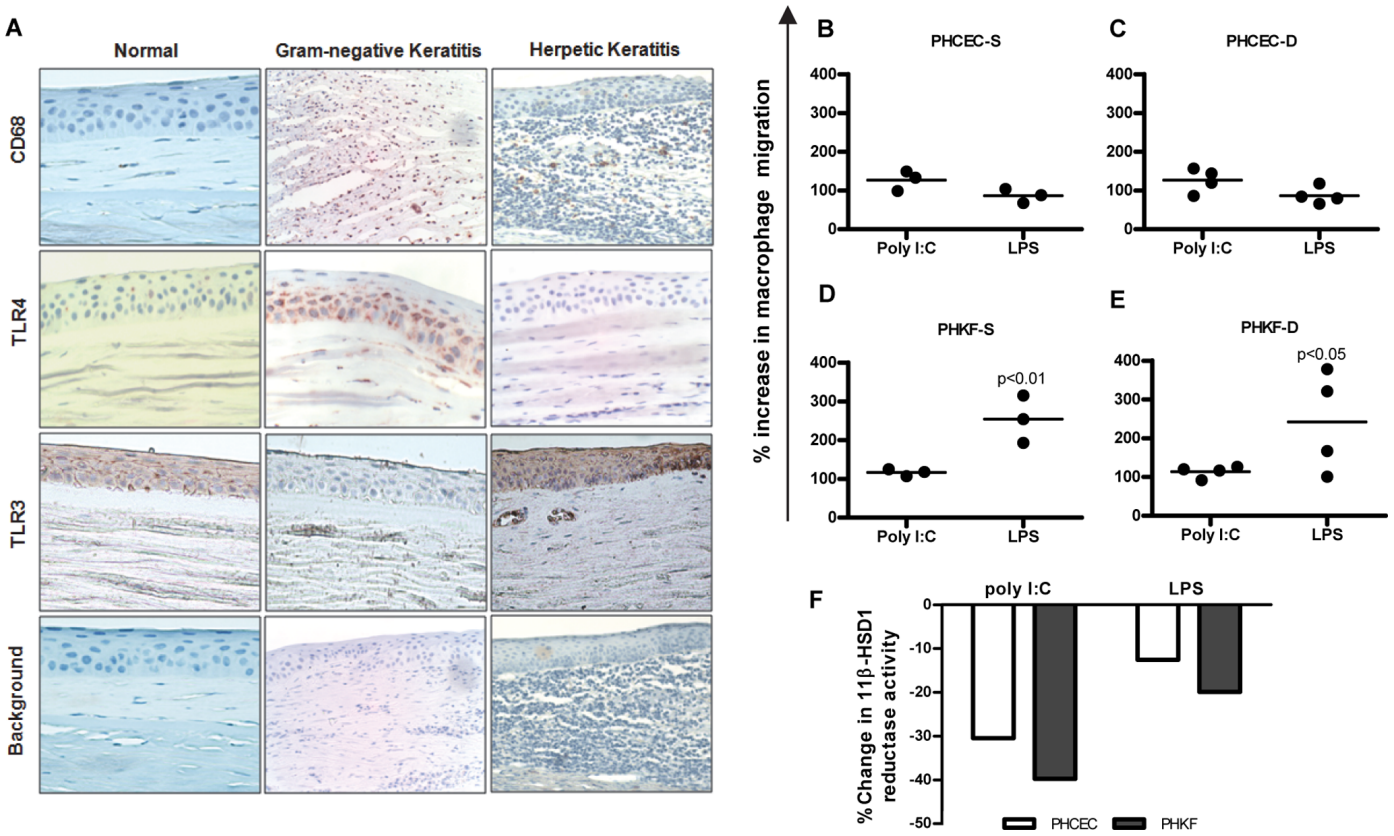
Macrophage infiltration in human keratitis. (A) Immunohistochemistry of the normal human central corneal epithelium showed no evidence of CD68 positive resident macrophages or basal TLR4 expression, although there was some basal TLR3 expression. Corneal stromal infiltration of CD68 positive cells is seen in both herpetic and gram negative keratitis associated with increased TLR3 and TLR4 expression in the corneal epithelium, respectively. (B–E) Migration assay showing culture supernatants of corneal cells having chemotactic potential on monocytes. Cell supernatants from PHCEC/PHKF stimulated with TLR3 (poly I∶C) or TLR4 (LPS) ligands for 16 h were tested for the ability to induce monocyte migration. ‘S’ denotes culture supernatants from PHCEC/PHKF cultures generated from 3 corneal donors, tested on a single allogenic PBMC donor. ‘D’ denotes 3 different PBMC donors subjected to culture supernatant from a single donor derived PHCEC/PHKF treated with TLR3 and TLR4 ligands. Data show that both LPS and Poly I∶C stimulation of corneal cells induce monocyte migration but LPS stimulation of PHKF has the greatest chemotactic potential. (Panels D and E). (F) Culture supernatants from experiments A–D (TLR3 and TLR4 induction of PHCEC/PHKF for 16 h) downregulates M1 macrophage 11β-HSD1 activity. Statistical analysis was carried using one-way ANOVA and comparisons were drawn with untreated control cells vs. TLR3/TLR4 treated cells.

The interplay between infiltrating monocytes/macrophages and corneal cells was examined. Immunohistochemistry of the normal human central corneal tissue showed no evidence of CD68^+ve^ resident macrophages, and only basal TLR3 or no TLR4 expression. Stromal infiltration of CD68^+ve^ positive cells was seen in both herpetic and gram-negative keratitis sections (**Figure 4A**), associated with TLR3 and TLR4 expression in the corneal epithelium, respectively. The response was most pronounced in gram-negative keratitis.

To determine if inflammatory cell infiltration into infected corneas was mediated by PHCEC and PHKF cytokines after TLR3 and TLR4 ligation, migration assays were performed. Supernatants from three different donor PHCEC cultures treated with TLR3 ligand poly I∶C, induced migration of human monocytes generated from (i) one PBMC donor ((127±19% (mean±SE) increase in migration compared with control **Figure 4B**: PHCEC-S), and (ii) with one culture supernatant from one corneal donor tested on 4 different monocyte donors (127±24% increase **Figure 4C**: PHCEC-D). A similar effect was seen when PHKF were stimulated by poly I∶C (**Figure 4D**: PHKF-S and - **Figure 4E**: PHKF-D (117±7% and 114±11% respectively)). Importantly, LPS (TLR4) stimulation of PHKF produced supernatants that induced potent monocyte migration in both experiments (**Figure 4D and E** 254±41% for PHKF-S and 242±10.8% for PHKF-D p<0.01) supporting immunohistochemistry data, while the effect on PHCEC was weaker (**Figure 4B and C** - 87±13% for PHCEC-S and 87±16% for PHCEC-D respectively). These results show a differential chemotactic potential to TLR induction between PHCEC and PHKF.

### Corneal cell supernatant control of macrophage cortisol production

As TLR stimulation of corneal cells induced differential rates of monocyte chemotaxis, we investigated whether the cumulative inflammatory response was a candidate for regulating macrophage intracrine cortisol bioavailability. There was a marked depletion of macrophage cortisol synthesis after TLR3 challenge of both PHCEC (−30%) and PHKF (−40%) (supernatants taken from the migration experiments described above), and after TLR4 challenge (PHCEC −10%; PHKF −20%) (**Figure 4F**). These data indicate that net cortisol biosynthesis is attenuated after activation of innate signaling pathways in the ocular surface.

### Relationship between glucocorticoid hormone metabolites in human ocular biofluids, in health and disease

Having demonstrated that corneal cells *in vitro* have 11β-HSD1 oxoreductase activity and that macrophage cortisol bioavailability was inhibited following incubation with TLR stimulated corneal cell supernatant, which could occur during chemotaxis, we sought to investigate whether the human OcS, *in vivo*, was capable of synthesizing cortisol from cortisone in healthy eyes and during active infective keratitis or putative TLR 3 mediated OcSD disease. We used a highly sensitive technique of LC/MS/MS and quantified GC metabolites in human ocular biofluid compartments as surrogates for OcS (tears) and intraocular (AqH) steroidogenesis, versus peripheral blood (serum).

#### (i) Normal healthy eyes

**Figure 5:**
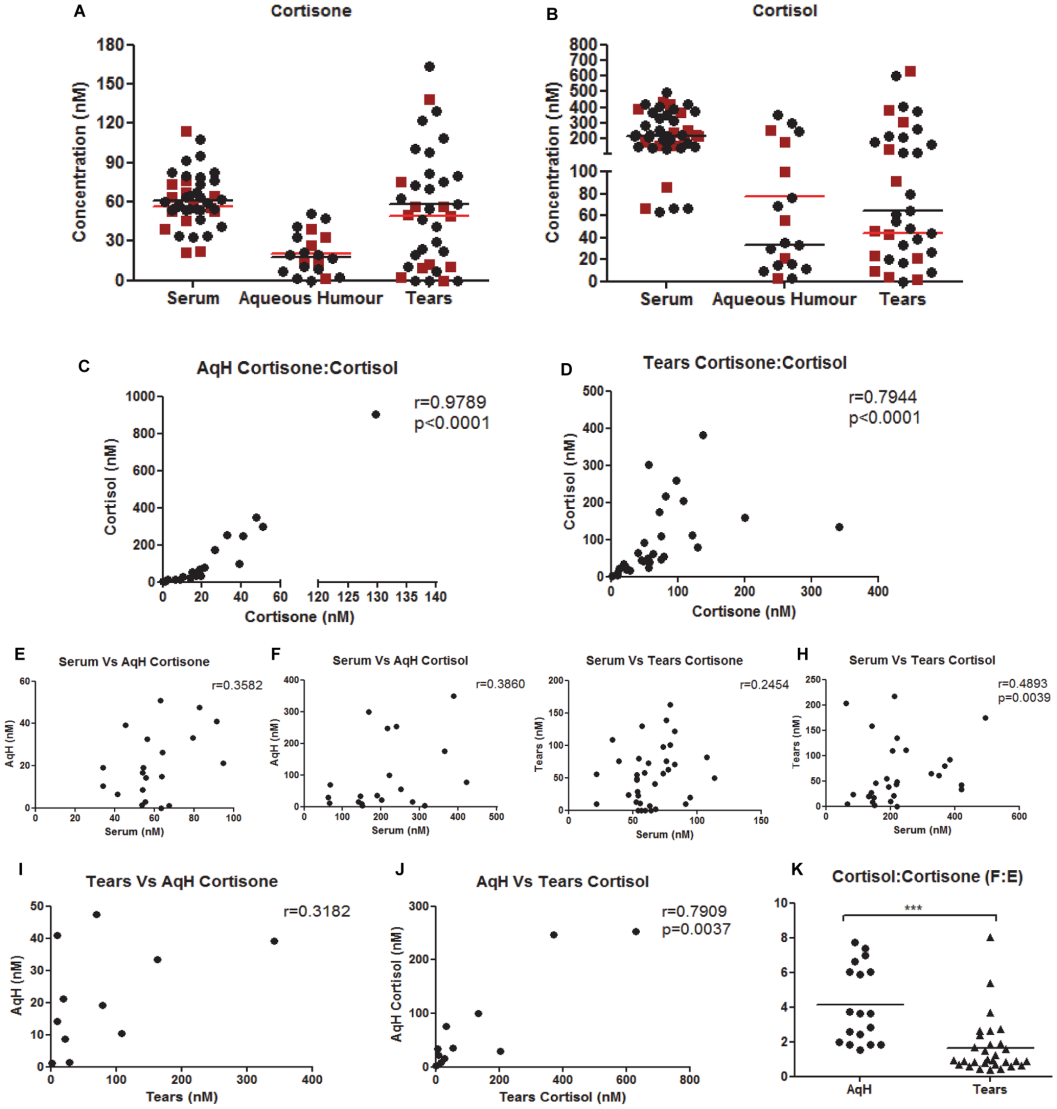
Hormones detected in human ocular biofluids versus serum. A) Cortisone and (B) cortisol levels in the tear film and AqH. There was no gender dependency for any of the analyte (• black circle, Male and (▪ red square, Female)). There was a positive correlation between cortisol and cortisone in both ocular biofluids (C–D), but concentrations were largely independent of those found in serum (E–H). There was an association between AqH and Tear film cortisone (I) and cortisol (J), and cortisol∶cortisone (F∶E) ratios were consistently higher in AqH versus the tear film (K).

Both cortisone and cortisol were detected in all three bio-fluid compartments in the proportion serum = tears>AqH and serum>tears = AqH, respectively (**Figure 5 A–B**). No gender difference was observed. Both ocular biofluids showed positive correlation between cortisone and cortisol levels, AqH (r = 0.98, p<0.0001, **Figure 5C**) and tears (r = 0.79, p<0.001, **Figure 5D**), but there was no correlation between serum cortisol and cortisone versus that found in tears or AqH (**Figure 5E–H**) indicating that simple diffusion from the peripheral circulation into the tear film and AqH was not likely to be the main source of these hormones. Cortisone and cortisol levels were consistently higher in tears versus AqH where a positive correlation was observed between tear cortisol vs AqH cortisol (r = 0.79, p<0.003) (**Figure 5I–J**), suggesting that the tear film may be the driver for AqH cortisol, delivering either active cortisol into the anterior chamber or substrate (cortisone) for intraocular 11β-HSD1. The cortisol∶cortisone ratio (a marker of 11β-HSD1 oxo-reductase activity) showed cortisol∶cortisone ratios in AqH (3∶1) exceeded those in tears (1∶1) in healthy individuals (**Figure 5K**). These data suggest greater 11β-HSD1 activity within the eye than on the surface of the eye in normal physiological states. There was no relationship between age or gender on cortisol or cortisone levels, or cortisol∶cortisone ratios, in each of the ocular bio-fluids (**Figure S4A (cortisol), S4B (cortisone), S4C (cortisol∶cortisone)**).

#### (ii) Infective keratitis and immune-mediated ocular surface disease

**Figure 6:**
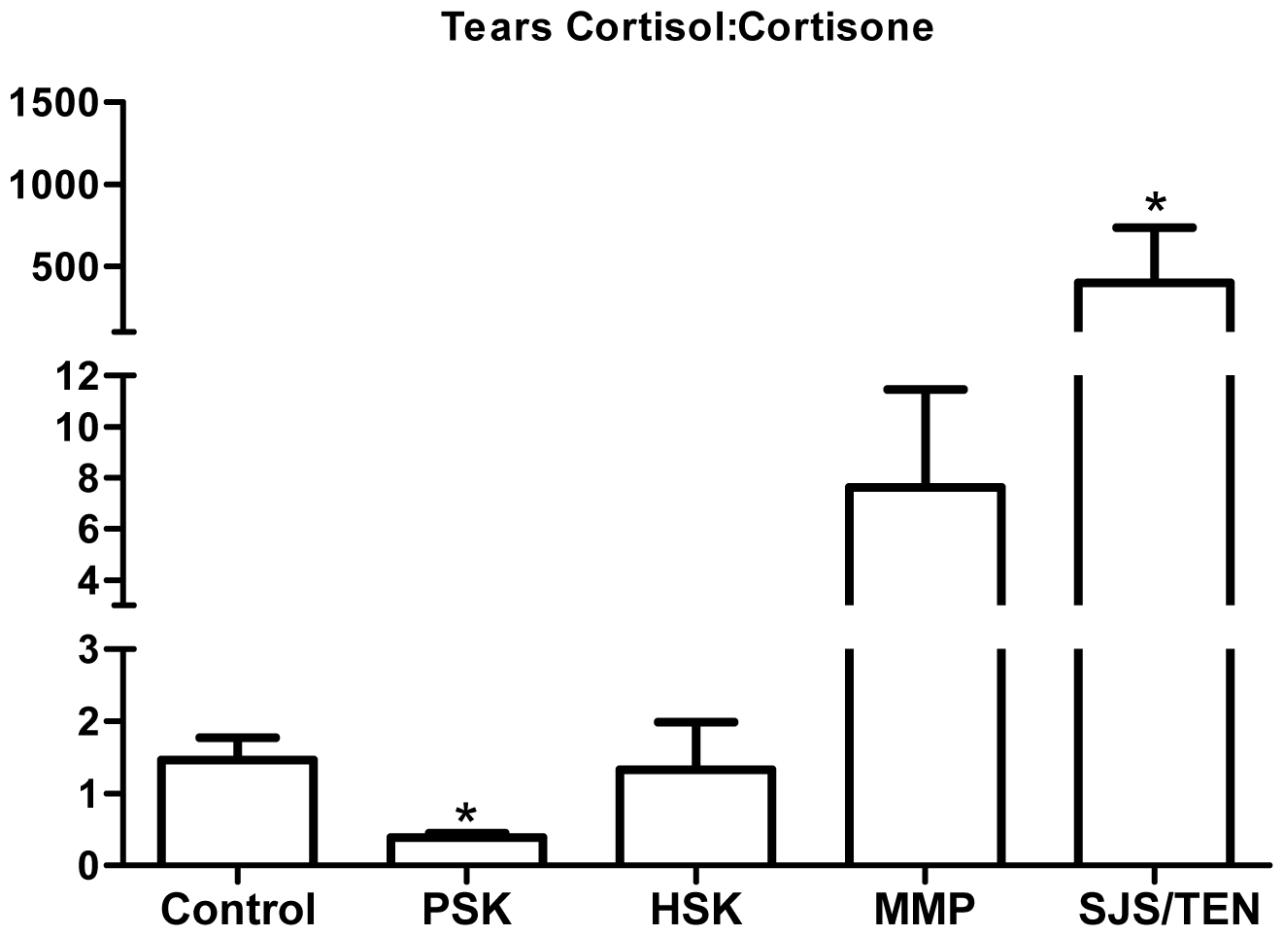
Ocular surface glucocorticoid bioavailability in health and disease. Evaluation of tear film glucocorticoid profiles as surrogate readouts of net ocular surface glucocorticoid bioavailability, defines cortisol depletion (reduced cortisol∶cortisone (F∶E ratio, mean±SE) during active untreated pseudomonas keratitis (PSK) but not herpes simplex keratitis (HSK), and amplification during chronic clinically quiescent immune-mediated disease (MMP, mucous membrane pemphigoid; SJS/TEN, Stevens-Johnson Syndrome/Toxic-epidermal Necrolysis). Statistical analysis performed using t-test with two-tailed Mann Whitney post -test. * = P<0.05.

Having established that the healthy human OcS is capable of generating cortisol from cortisone *in vivo*, and that *in vitro* induction of TLR3 and TLR4 in PHCEC and PHKF triggered pro-inflammatory cytokine production with subsequent macrophage migration and abrogation of immune-cells cortisol biosynthesis, we sought to establish whether the tear film glucocorticoid profile was altered in patients with untreated herpetic and pseudomonas keratitis presenting to the emergency room, and those patients with chronic immune-mediated ocular surface inflammatory disease. These data showed that the mean (±SEM) free cortisol in healthy volunteer tear film 85.6(0–1306.2) nM/L was lower than in patients with acute herpes simplex keratitis (288.1(136.5–434.1) nM/L) and pseudomonas keratitis (223.0 (154.9–418.6) nM/L). These data equated to cortisol∶cortisone ratios of 1.33(0.39–131.3) in the healthy tear film versus 0.33(0.0–0.50) (p<0.01) in patients with pseudomonas keratitis and 1.42(0.12–6.44) (p = 0.42) in herpes simplex keratitis (**Figure 6**), confirming an attenuation of OcS cortisol biosynthesis (and 11β-HSD1 activity) *in vivo* during active OcS pseudomonas infection (supporting *in vitro* data (**Figure 4E**)). By contrast, cortisol∶cortisone ratios were increased in patients with chronic (putative TLR 3 regulated) immune-mediated OcS disease states (OcMMP, 9.05(0.9–20.7); SJS/TEN, 113.8(11.73–1070.0 (p<0.01) (**Figure 6**).

## Discussion

In the human eye, the ocular surface provides the first line of defense to potential exogenous triggers, but is also a target for autoimmune disease. The surface is a non-keratinized mucosa with a unique avascular component known as the cornea, critical for refracting light and for sight. The cornea is considered to be an immune privileged site exhibiting both immunological ignorance, but also a range of active immunosuppression mechanisms [29] that infer a 90% 5 year survival rate of low risk corneal allografts [30], [31]. Several pathways have been purported to be involved in the immune-privileged status of the eye including a range of systemic and neural factors, in addition to local intraocular mechanisms such as soluble immunosuppressive agents in the aqueous humour [α-melanocyte stimulating hormone (αMSH), vasoactive intestinal peptide (VIP), transforming growth factor-β2 (TGF-β2), IL-1Rα, somatostatin, cortisol][32]–[34] or cell surface molecules (CD95-L, B7H1, MHC Class Ib, CD46, CD55, CD59) [35].

Adrenal hormones are known to be regulators of ocular surface health and dry eye induced inflammation [36]–[40]. Our previous work has indicated that endogenous production of GCs mediated by the isozyme 11β-HSD1 may also be a contributing factor, specifically ocular surface renewal and putative barrier function [3], [15]. 11β-HSD1 is involved in several disease processes including insulin resistance, osteoporosis, obesity, idiopathic intracranial hypertension, glaucoma, orbital adipogenesis, inflammation and immune cell function [3], [28], [32], [41]–[43].

While a small resident population of myeloid cells populate the central cornea [44], critical to clearing infection is the recruitment of phagocytes (macrophages and iDCs) to the site of infection and these cells too, appear to be mediated in part, by local cortisol. There is an enhanced ability of myeloid-derived immune cells to produce cortisol after TLR-induced maturation signals. This is associated with an increased expression of MHC class I and class II, CD40L and synthesis of pro-inflammatory cytokines, leading to inflammation at the site of infection [20]. Conversely, maturation of iDCs induced by CD40 ligation not only leads to a fall in the ability of DCs to produce cortisol, differentiation of DCs from monocyte precursors is inhibited by physiological concentrations of cortisone, indicating that inactive GC acts as an autocrine-negative regulator of DC maturation thereby imposing a checkpoint on differentiation of monocytes to mature DCs [24], [25].

In this study, we examined the tissue-specific cortisol regulation by human corneal cells in the context of immune responses to TLR ligation, and how this could have impact on clinical disease. Both PHCEC and PHKF cells were shown to express major components of the glucocorticoid pre-receptor signaling pathways consistent with earlier human and rabbit studies [3], [14]. These cells were capable of producing cortisol, although the rate of conversion was less than that of M1 and M2 macrophages. In our study, TLR ligation *in vitro* did not alter cortisol bioavailability but elicited cell-specific cytokine and chemokine release, including TNFα only after TLR 1, 4, 7, and 9 stimulation of PHCEC; and CCL2, IL-6, CXCL8, G-CSF after TLR 1–8 stimulation of PHKF. Cytokine induction however, was regulated by the presence of synthetic (dexamethasone) and natural (cortisol) GCs in PHKF only, but only TNFα in PHCEC. This dichotomy is of interest, and the relative resistance of PHCEC to glucocorticoids is the focus of future study.

Stimulation of PHCEC with TLR ligands showed that only poly I∶C could induce multi-cytokine synthesis. The range of molecules produced, include proinflammatory cytokines IL-12 and IFNγ indicative of a Th1 mediated adaptive response, chemokines CCL3, CCL4, and CCL5 that are associated with mononuclear cell recruitment, and IFNβ that has both antiviral and immunosuppressive function. There is a particularly strong increase in CXCL10 production after ligation of TLR 3 in both cell types and also TLR4 in HKF. This is of interest as this cytokine has pleiotropic function including angiostasis due to its lacking an ELR motif (Glu-Leu-Arg tripeptide sequence). In CXCL10 deficient mice, an increase in liver fibrosis is seen due to inhibition of natural killer cells (NK) which target hepatic stellar cells [45]. In murine corneal infection with Herpes simplex virus, depletion of NK cells leads to loss of viral control [46]. As TLR3 recognizes double-stranded RNA these data support an inductive role for PHCEC in response to infection or possibly TLR3 related immune-mediated disease e.g. SJS-TEN, dry eye disease states, may induce an increase in CXCL10 and a reduction in NK cells exacerbating infection or persistence of inflammation. With the exception of TNFα, the failure of other TLR ligands to induce cytokines by PHCEC has been suggested to be due to the intracellular expression of TLR2 and TLR4 in PHCEC, receptors that are normally expressed on the cell surface [47]. It is also possible that PHCEC do not have the co-receptors necessary for TLR signaling, although CD14 expression has been reported [48]. This relative inertness of the corneal epithelium may be a contributory factor to barrier function, and it is possible that breaches of this barrier are required to trigger resident stromal keratocytes to differentiate into an activated fibroblast phenotype with subsequent wider cytokine response, as shown in our study. Activated fibroblasts may therefore represent sentinels for ocular surface inflammation. Regardless, the stimulation of TLR3 strongly suggests and supports the concept that the human OcS produces a vigorous immune response possibly underpinning reactivation of viral keratitis and exacerbations of inflammation in chronic OcS immune-mediated disease [18], [21]–[23].

Responses in PHKF to TLR ligands were inhibited by the addition of exogenous glucocorticoids. Unlike monocyte–derived cells [24], [49]. TLR stimulation had no effect on cortisol biosynthesis by PHCEC or PHKF. By comparison, blood-derived macrophages, particularly M1 cells showed 3-fold greater production of cortisol compared to ocular cells. These data suggest that infiltrating inflammatory (M1) macrophages are capable of controlling not only their own inflammatory processes, but also ocular surface cytokine production via paracrine cortisol control. While endogenous cortisol has been shown in several studies to down-regulate MHC class II, co-stimulatory molecules (CD80, CD86) and the ability to activate allogeneic cells, inhibition of endogenous cortisol can also lead to a reduction in IL-1 and TNFα. A recent study has shown that IL-17 may orchestrate resolution of innate inflammation, whereas IFN-γ and IL-4 may represent major determinants of IL-10 and glucocorticoid resistance [50].

The potential role of cortisol produced by corneal cells versus that produced by infiltrating macrophages is of considerable interest [51]–[54]. The differing response to TLR induction between ocular surface epithelial cells and macrophages may be an element of silencing induced by coexistent protective commensal bacteria, a mechanism seen in the gut vital intestinal homeostasis [55]. Breaches in intestinal epithelial cell cross-communication with gut flora provide the basis of inflammatory bowel disease and this may be relevant in driving ocular surface disease pathogenesis.

Using the novel technique of LC/MS/MS we have been able to detect cortisol and cortisone levels in human ocular bio-fluids as functional readouts of tissue specific regulation of cortisol. Overall, cortisol levels were greater than those reported in other bio-fluids such as CSF [28], saliva [56] and skin microdialysis [57]. Cortisol concentrations at the ocular surface consistently exceeded those in the intraocular aqueous humor amongst healthy human subjects in a consistent pattern of expression (serum>tears>AqH) with no gender variations observed. Correlations of cortisol in ocular bio-fluids versus those found in serum, confirmed that ocular cortisol was potentially independent of circulatory cortisol. By contrast, intraocular cortisol and cortisone correlated with that on the ocular surface (consistently tears>AqH). Cortisol∶cortisone (F∶E) ratios (an *in viv*o biomarker of 11β-HSD1 enzyme activity) were greater within aqueous humor than in the tear film. These data indicate that the OcS cortisol-cortisone shuttle may be the driver for intraocular cortisol, either by delivering cortisol directly into the anterior chamber of the eye or substrate for 11β-HSD1. In patients with acute presentation of pseudomonas keratitis, the net ocular surface cortisol∶cortisone ratios were significantly reduced, suggesting either an abrogation of cortisol is required to allow inflammatory cell migration to eradicate infection, or an increased utilization of cortisol in an acute bacterial disease state. These data supported our *in vitro* findings that demonstrated a reduction in macrophage 11β-HSD1 oxo-reductase activity when challenged by a cytokine cocktail produced by either PHCEC or PHKF induced by TLR4 ligation. It would be interesting to interrogate longitudinal tear film glucocorticoid profiles during clinical disease course, to determine whether specific profile patterns correlate with the sterilization and healing phases of infective corneal disease. Although there appeared to be no change in net tear film cortisol∶cortisone ratios in patients with untreated herpes simplex keratitis, there was a marked intensification of cortisol compared to cortisone in patients with chronic immune-mediated disease states not using local or systemic therapeutic glucocorticoids. TLR3 is an integral player in chronic SJS-TEN [23], experimental inflammatory dry eye [19] and hypersensitivity responses in the conjunctiva [58]. While the local regulation of cortisol is clearly important in physiological and innate immune responses in the eye, the role is multifaceted and the mechanisms underpinning therapeutic glucocorticoid use is highly complex. The data though intriguing is based on small numbers of patients with each disease and further investigation is necessary to explore these data further, with the potential for longitudinal studies an important consideration

**Figure 7:**
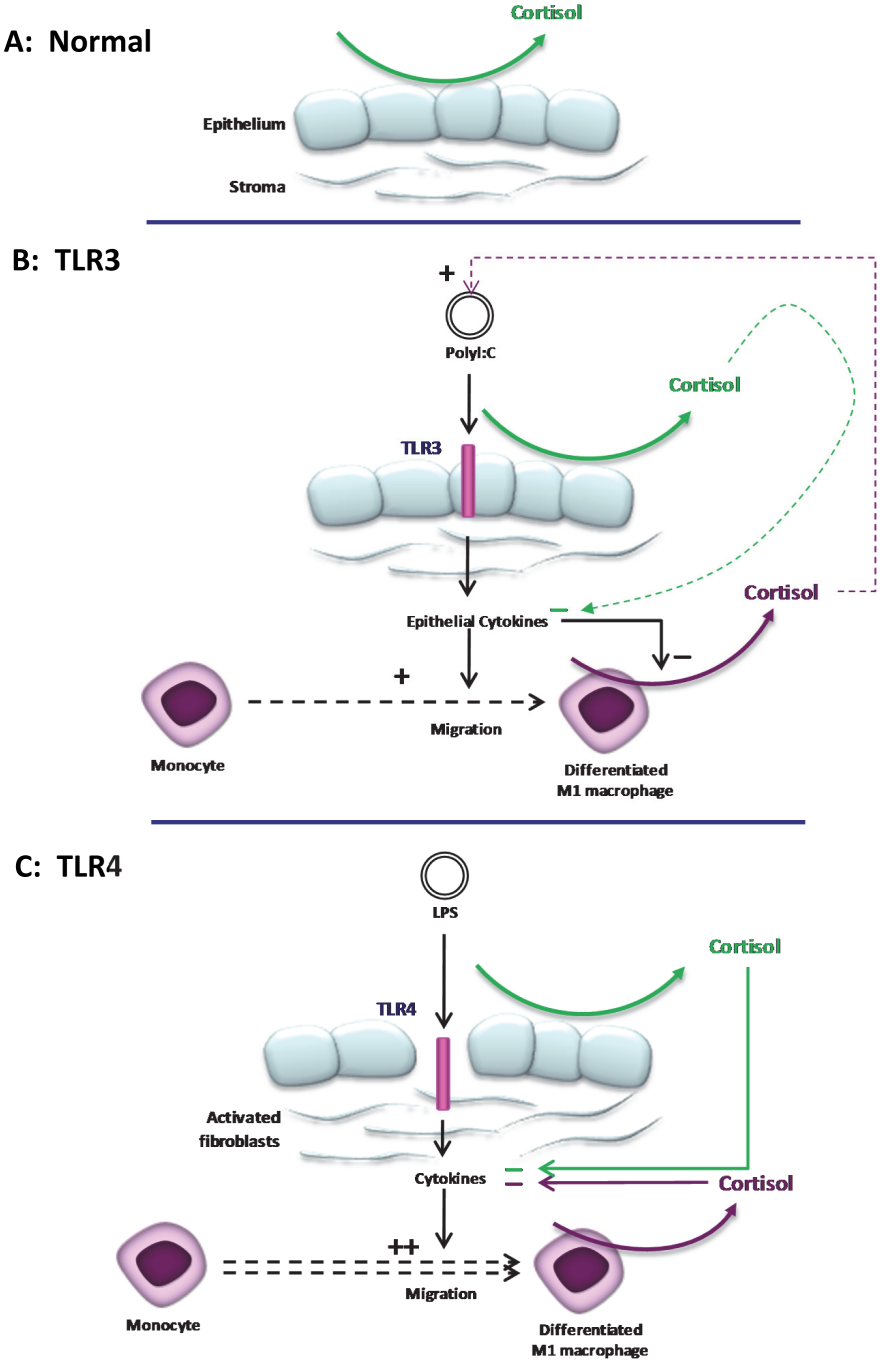
Putative interaction of TLR signaling and local regulation of cortisol in the human cornea. (A) Under physiological conditions, autocrine synthesis of cortisol in corneal epithelial cells contributes to the immunoprotection of the ocular surface mucosa. During induction of ocular surface TLRs cytokines are released in a ligand and cell specific manner. (B) On TLR3 ligation such as chronic immune -mediated disease e.g. SJS-TEN, synthesis of a diverse spectrum of cytokines primarily from the corneal epithelial cells, induces weak monocyte chemotaxis and differentiation to M1 macrophages. These cytokines potently attenuate M1 cortisol biosynthesis leading to a net reduction of ocular surface cortisol levels, promoting recruitment of inflammatory cells necessary for resolving the initial trigger. By contrast, on TLR4 ligation (C) activation of keratocytes to a fibroblast phenotype, form the first line of defense producing chemokines that potently induce monocyte migration to the site of infection for rapid eradication of bacterial invasion. Attenuation of M1 cortisol production is less pronounced and this facilitates resolution of the inflammatory response, limiting tissue damage thereby preserving optical clarity (and sight).

In summary, we have shown that intracrine cortisol biosynthesis in human corneal cells is independent of TLR activation and is likely to afford immuno-protection under physiological conditions (**Figure 7A**). During microbial or immune-mediated challenge, release of cytokines from these cells, trigger migration of monocytes and differentiation to macrophages that in turn are capable of generating large quantities of cortisol that contribute to resolution of the local inflammatory response thereby maintaining optical clarity (**Figures 7B and C**). Nevertheless, *in vivo*, the net OcS cortisol measured in the tear film is dependent on the nature of the OcS disease and a reduction in acute infection or an increase during chronic immune-mediated disease, is observed. These data indicate that OcS generation of cortisol contributes to ocular mucosal innate responses, but its role is complex and dependent on the cell type and nature of the underlying immunological challenge.

## Supporting Information

Figure S1 **Expression of TLRs in Primary Corneal Cells.** PHCEC expressed TLR1, TLR2, TLR3, TLR4 and TLR10 mRNA, while PHKF expressed mRNA for TLR 1–4, and TLR9-10. GAPDH was used as a housekeeping gene and distilled water (DW) alone as the negative control.
(TIF)

Figure S2 **Cytokine Expression after TLR Stimulation of Primary Corneal Cells.** Multiplex bead ELISA analysis (Plex-30) of cytokine production in PHCEC and PHKF in response to TLR 1–9 stimulation for 16 hours is shown. Cytokines analysed included: IL-1β, IL-1Rα, IL-2, IL-4, IL-5, IL-6, IL-7, IL-8, IL-9, IL-10, IL-12(p70), IL-13, IL-15, IL-17, Eotaxin, Basic FGF, G-CSF, GM-CSF, IFN-γ, CXCL-10, CCL2, CCL3, CCL4, PDGF-BB, CCL5, TNF-α, VEGF. TNFα increased after TLR 1, 4, 7, and 9 stimulation of PHCEC and IL6 after TLR 3. (B) PHKF were variably responsive to TLRs 1–8 producing non-significant induction of G-CSF, CCL2, IL-6, IL-8, CXCL10 and CCL5. Values =  Mean+SE, n = 3, Statistical analysis was carried using One-way ANOVA and comparisons were drawn with untreated control Vs TLR stimulated cells.
(TIF)

Figure S3 **Effect of Glucocorticoids on the Production of Corneal Cell Cytokine Production.** Both Dexamethasone (▪, grey square) and cortisol (▪, black square) had no effect on the production of the cytokines (VEGF, CCL2, IL-6, IL-12, GCSF, IFN-Y,CXCL10, IL-8) after TLR 3 and 4 stimulation in PHCEC. However, for PHKF, the cytokines MCP-1 and Il-6 were over the detection limit and TNF-α and IL-12 were below the detection limit and the effect of cortisol or dexamethasone could not be verified on these cytokines. (Values =  Mean+SE, n = 3 normalized to no Cortisol/Dexamethasone □, white).
(TIF)

Figure S4 **Correlation studies of glucocorticoid bioavailability in the human eye with age.** There was no correlation between cortisol and cortisone and their ratios in both ocular biofluids (Tears or AqH) with age or gender (A–C).
(TIF)

Table S1 **Oligonucleotide sequences of PCR primers.**
(DOC)
