# Supplementary figures and images for: Cortisol Biosynthesis in the Human Ocular Surface Innate Immune Response
Source: PLoS One. 2014 Apr 15;9(4):e94913. doi: 10.1371/journal.pone.0094913 (PMC3988113; doi:10.1371/journal.pone.0094913)

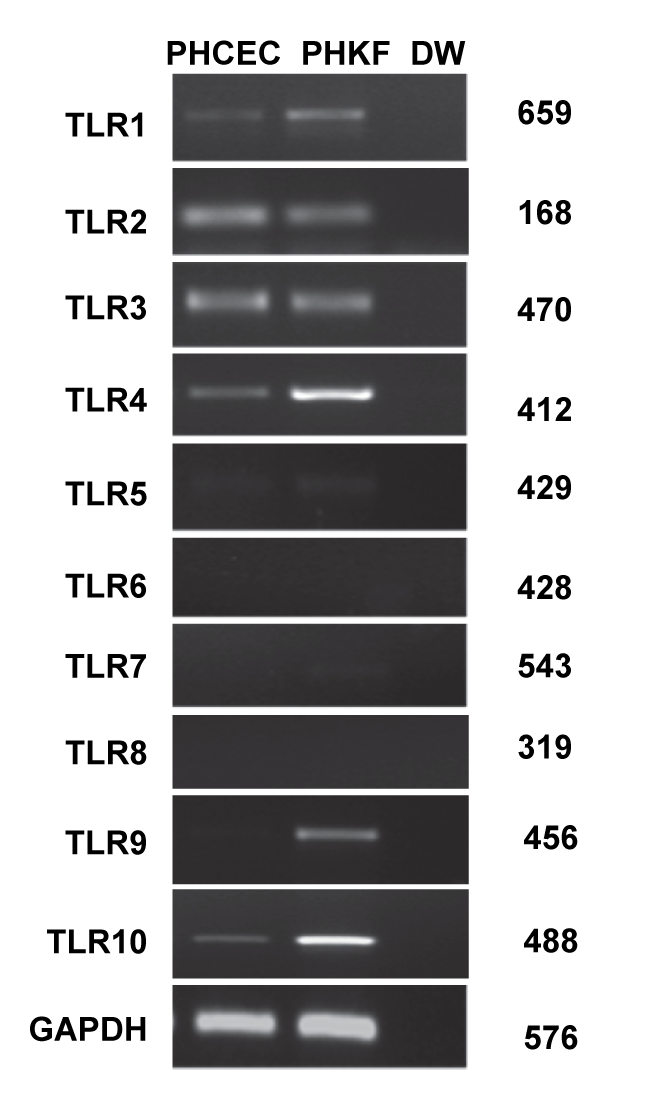

Supplement: Figure S1 — Expression of TLRs in Primary Corneal Cells. PHCEC expressed TLR1, TLR2, TLR3, TLR4 and TLR10 mRNA, while PHKF expressed mRNA for TLR 1–4, and TLR9-10. GAPDH was used as a housekeeping gene and distilled water (DW) alone as the negative control. (TIF) [file pone.0094913.s001.tif]

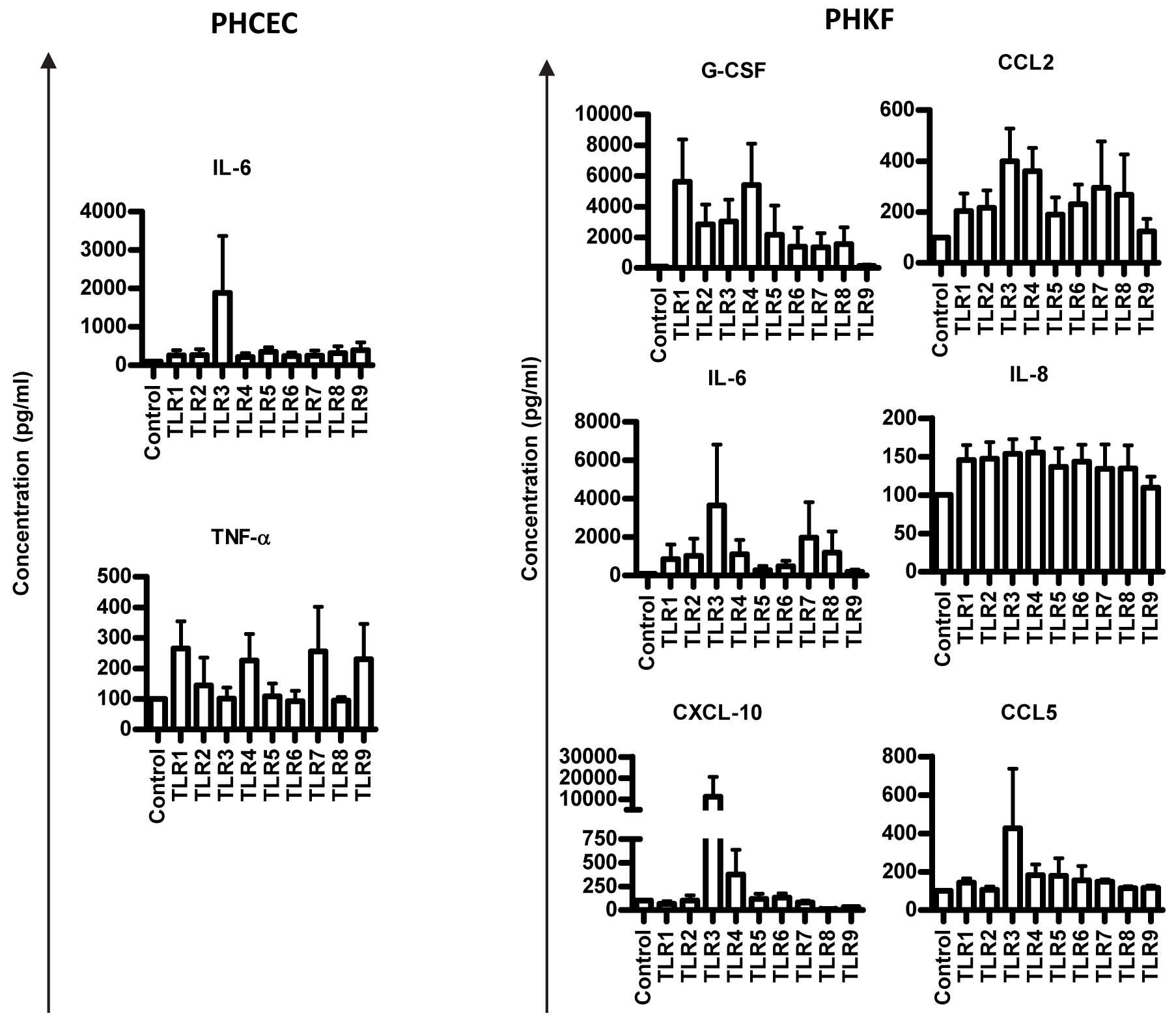

Supplement: Figure S2 — Cytokine Expression after TLR Stimulation of Primary Corneal Cells. Multiplex bead ELISA analysis (Plex-30) of cytokine production in PHCEC and PHKF in response to TLR 1–9 stimulation for 16 hours is shown. Cytokines analysed included: IL-1β, IL-1Rα, IL-2, IL-4, IL-5, IL-6, IL-7, IL-8, IL-9, IL-10, IL-12(p70), IL-13, IL-15, IL-17, Eotaxin, Basic FGF, G-CSF, GM-CSF, IFN-γ, CXCL-10, CCL2, CCL3, CCL4, PDGF-BB, CCL5, TNF-α, VEGF. TNFα increased after TLR 1, 4, 7, and 9 stimulation of PHCEC and IL6 after TLR 3. (B) PHKF were variably responsive to TLRs 1–8 producing non-significant induction of G-CSF, CCL2, IL-6, IL-8, CXCL10 and CCL5. Values = Mean+SE, n = 3, Statistical analysis was carried using One-way ANOVA and comparisons were drawn with untreated control Vs TLR stimulated cells. (TIF) [file pone.0094913.s002.tif]

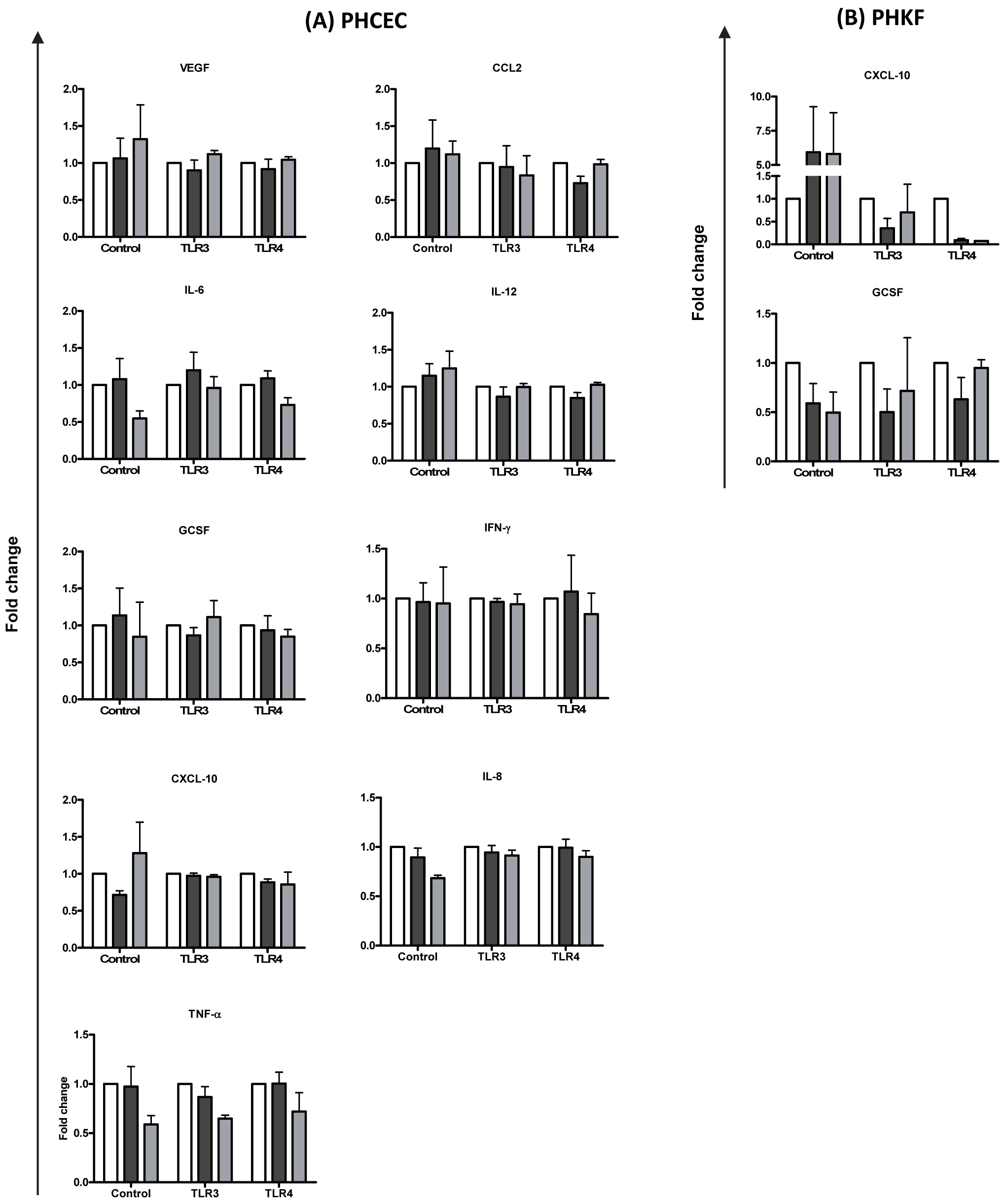

Supplement: Figure S3 — Effect of Glucocorticoids on the Production of Corneal Cell Cytokine Production. Both Dexamethasone (▪, grey square) and cortisol (▪, black square) had no effect on the production of the cytokines (VEGF, CCL2, IL-6, IL-12, GCSF, IFN-Y,CXCL10, IL-8) after TLR 3 and 4 stimulation in PHCEC. However, for PHKF, the cytokines MCP-1 and Il-6 were over the detection limit and TNF-α and IL-12 were below the detection limit and the effect of cortisol or dexamethasone could not be verified on these cytokines. (Values = Mean+SE, n = 3 normalized to no Cortisol/Dexamethasone □, white). (TIF) [file pone.0094913.s003.tif]

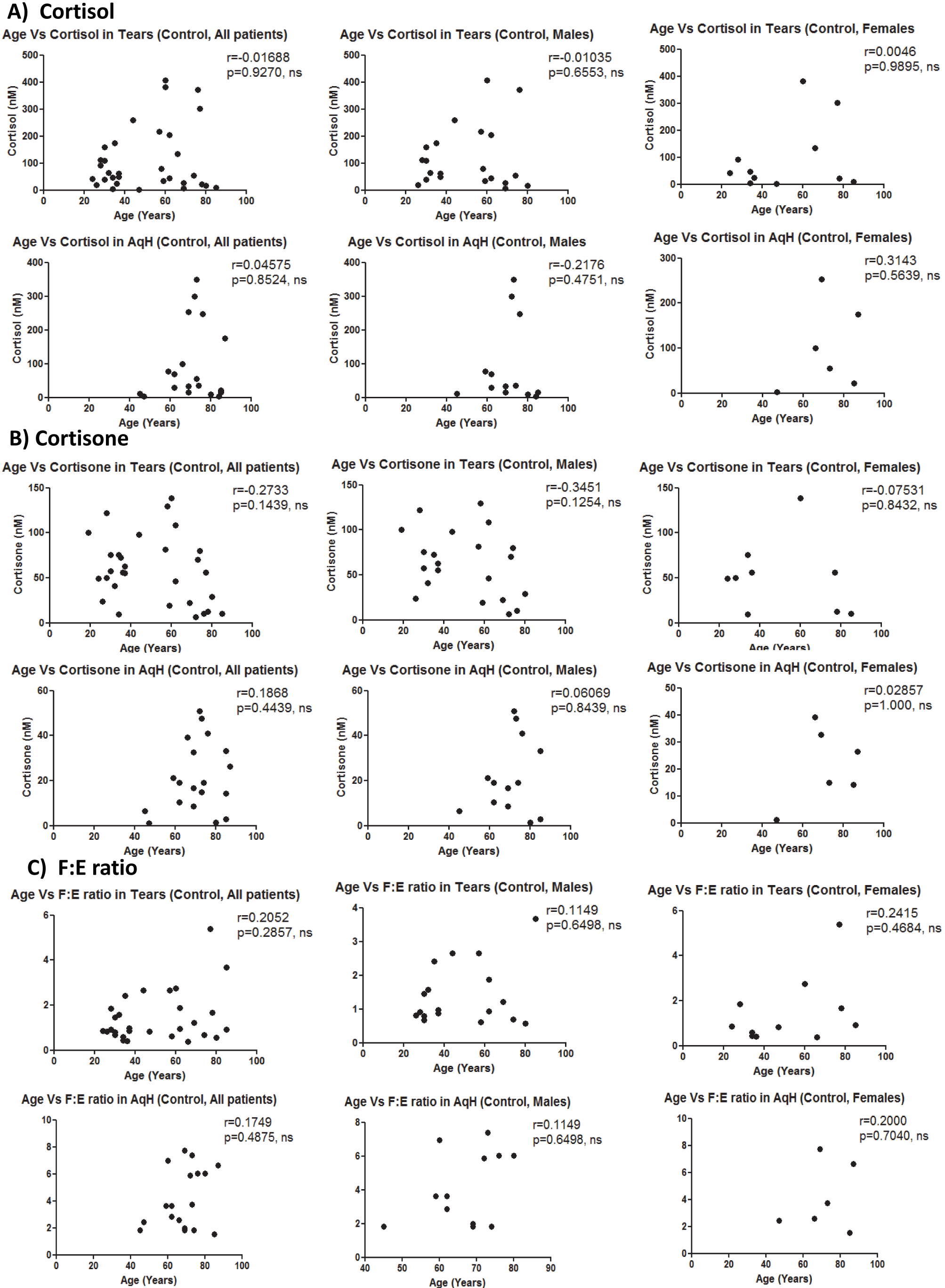

Supplement: Figure S4 — Correlation studies of glucocorticoid bioavailability in the human eye with age. There was no correlation between cortisol and cortisone and their ratios in both ocular biofluids (Tears or AqH) with age or gender (A–C). (TIF) [file pone.0094913.s004.tif]
