# Supplementary material for: Cortisol Biosynthesis in the Human Ocular Surface Innate Immune Response
Source: PLoS One. 2014 Apr 15;9(4):e94913. doi: 10.1371/journal.pone.0094913 (PMC3988113; doi:10.1371/journal.pone.0094913)
Supplement: Table S1 — Oligonucleotide sequences of PCR primers. (DOC) [file pone.0094913.s005.doc]

# Supporting Table

## Table S1. Oligonucleotide sequences of PCR primers

| **Gene** | **Direction** | **Primer sequence** | **Product size (bp)** | **Annealing temperature (°C)** |
| --- | --- | --- | --- | --- |
| **11β-HSD1** | Forward  Reverse | 5’ ACC AGA GAT GCT CCA AGG AA 3’  5’ ATG CTT CCA TTG CTC TGC TT 3’ | 411 | 60 |
| **H6PD** | Forward  Reverse | 5’ AGA AGC GAG ACA GCT TCC AC 3’  5’ GCT GCT GGG AAA AGA ACA AC 3’ | 603 | 60 |
| **GRα** | Forward  Reverse | 5’ TCG ACC AGT GTT CCA GAG AAC 3’  5’ TTT CGG AAC CAA CGG GAA TTG 3’ | 693 | 60 |
| **11β-HSD2** | Forward  Reverse | 5’ TGG AGG TGA ATT TCT TTG GC 3’  5’ GGA TTC TTT AGG CCA GGG TC 3’ | 773 | 55 |
| **MR** | Forward  Reverse | 5’ AAC TTG CCT CTT GAG GAC CAA 3’  5’ AGA ATT CCA GCA GGT CGC TC 3’ | 450 | 60 |
| **18S** | Forward  Reverse | 5’ GTT GGT GGA GCG ATT TGT CT 3’  5’ GGC CTC ACT AAA CCA TCC AA 3’ | 397 | 55 |
| **TLR1** | Forward  Reverse | 5’ acc aag ttg tca gcg atg tgt t 3’  5’ gat tgt ccc ctg ctt tta ttg a 3’ | 659 | 61 |
| **TLR2** | Forward  Reverse | 5’ gag tga gtg gtg caa gta tga 3’  5’ ggg cca ctc cag gta ggt ct 3’ | 168 | 61 |
| **TLR3** | Forward  Reverse | 5’ tcc caa gcc ttc aac gac tg 3’  5’ tcc tga aag ctg gcc cga aaa 3’ | 470 | 61 |
| **TLR4** | Forward  Reverse | 5’ tgc ggg ttc tac atc aaa 3’  5’ cca tcc gaa att ata aga aaa 3’ | 412 | 55 |
| **TLR5** | Forward  Reverse | 5’ ctc ctt tga tgg ccg aat agc 3’  5’ ccc aaa tga agg atg aag gta 3’ | 429 | 61 |
| **TLR6** | Forward  Reverse | 5’ caa ggc cct gcc cat ctg taa 3’  5’ ttg ggc caa aga aat tga aag 3’ | 428 | 61 |
| **TLR7** | Forward  Reverse | 5’ ccc cag cgt cct ttc aca ga 3’  5’ cga ggg caa ttt cca ctt agg 3’ | 543 | 61 |
| **TLR8** | Forward  Reverse | 5’ atg cgt gcc ttg tga tgg tg 3’  5’ gca atg ccc gta gag aca aaa 3’ | 319 | 61 |
| **TLR9** | Forward  Reverse | 5’ cta caa ccg cat cgt caa ac 3’  5’ atc gag tga gcg gaa gaa ga 3’ | 456 | 61 |
| **TLR10** | Forward  Reverse | 5’ acc cca gcc aca acg aca c 3’  5’ atc acg caa aag aac cca gaa 3’ | 488 | 61 |
|  |  |  |  |  |
|  |  |  |  |  |
|  |  |  |  |  |

Oligonucleotide sequences of PCR primers, their annealing temperatures and PCR product sizes (bp).
